# Implementing the learning health system paradigm within academic health centers

**DOI:** 10.1002/lrh2.10367

**Published:** 2023-04-13

**Authors:** Douglas Easterling, Anna Perry, David Miller

**Affiliations:** ^1^ Department of Social Sciences and Health Policy Wake Forest University School of Medicine Winston‐Salem North Carolina USA; ^2^ Wake Forest Clinical and Translational Science Institute Winston‐Salem North Carolina USA; ^3^ Department of Internal Medicine Wake Forest University School of Medicine Winston‐Salem North Carolina USA

**Keywords:** academic health center, academic medicine, implementation science, learning health system, organizational culture

## Abstract

**Introduction:**

The learning health system (LHS) concept represents a bold innovation that combines organizational learning, strategic analysis of patient data, stakeholder engagement and the systematic translation of research into practice – all in service of improving the quality of health care delivered across the organization. This innovation has been diffused and widely adopted by healthcare organizations over the past 15 years, but academic health centers (AHCs) have been slower on the uptake. The irony is that AHCs have the resources (e.g., trained researchers, sophisticated clinical data systems, informatics infrastructure) that are necessary to do the highest‐quality and most impactful LHS work.

**Methods:**

Based on a review of publications describing how AHCs have implemented LHS work, as well as the authors' direct experience promoting the adoption of the LHS paradigm at Atrium Health Wake Forest Baptist (AHWFB), we:identify a set of factors that have inhibited broader adoption of the LHS paradigm among AHCs; distinguish between the forms of LHS work that are consistent and inconsistent with the mission of AHCs; and offer recommendations for broader adoption and fuller implementation of the LHS paradigm.

**Results:**

The LHS paradigm represents an expansion of the scientific paradigm which serves as the foundation of research enterprise within AHCs. Both paradigms value rigorous studies of new treatments and practices, including pragmatic clinical trials. The LHS paradigm also places a high value on quality improvement studies, organizational learning, and the translation of research findings into improved patient care and operations within the local health system. The two paradigms differ on the origin of the research question, i.e., a pressing patient‐care issue facing the health system versus the investigator's own research interests. Academic researchers have been disincentivized from pursuing at least some forms of LHS research. However, a growing number of AHCs are finding ways to integrate the LHS paradigm into their research enterprise, either by providing research faculty with institutional funding to cover their effort on studies that address the health system's priority issues, or by establishing an institute dedicated to LHS research.

**Conclusions:**

The LHS paradigm is a disruptive intervention for AHCs, one that was initially resisted but is increasingly being embraced. AHCs are developing strategies for conducting LHS research, typically in parallel to the more traditional biomedical science that is core to academic medicine. Full implementation of the LHS paradigm will require further alignment between LHS and science, including a shift in the criteria for promotion and tenure to support those researchers who choose to focus on the pressing issues facing the health system.

For the past 15 years, the National Academy of Medicine (NAM) and the Agency for Healthcare Research and Quality (AHRQ) have encouraged healthcare organizations to become “learning health (or healthcare) systems” (LHS) as a means of accelerating both the translation of research into practice and the development of interventions that will improve patient care and outcomes.^1^, ^2^, ^3^ The LHS concept calls for healthcare organizations to be more systematic and data‐driven in generating and utilizing knowledge to improve the quality and value of the care they deliver, while also stimulating innovation.^4^

The LHS concept (originally framed as “learning healthcare systems”) first gained visibility at a 2006 workshop organized by NAM's predecessor, the Institute of Medicine (IOM).^5^ A handful of large health systems including Group Health Cooperative, Geisinger Health and Kaiser Permanente were centrally involved in translating the LHS concept into specific learning‐oriented practices, as well as identifying the organizational structures, policies and structures that are needed to support LHS work.^6^, ^7^, ^8^ By publishing articles that described their approaches, these health systems contributed to more widespread dissemination of LHS. In recent years, the LHS concept has been adopted and implemented by a much broader set of health systems, including nation‐wide health systems in countries such as Switzerland.^9^

## 1 ADOPTION OF LHS AMONG ACADEMIC HEALTH CENTERS

It is noteworthy that large academic health centers (AHCs) were not among the early adopters of LHS. One explanation is that AHCs had extensive research enterprises in place prior to 2006 and thus did not view LHS as an innovation. In contrast, health systems without an academic research enterprise were venturing into new territory, especially as their clinical data became more accessible and analyzable with the transition to electronic health records. AHCs may have also resisted the LHS concept because it emphasizes “applied” or “institutional” research over basic science and generalizable findings.

In a 2014 *JAMA* Viewpoint piece, Grumbach, Lucey, and Johnston argued that AHCs have placed too little emphasis on the translation of research findings into better care‐delivery practices, which they view as an essential aspect of a learning health system.^10^ They called for AHCs to bring their research and educational enterprises into direct alignment with the clinical enterprise under a single mission: “to improve health and health care through advancing, applying and disseminating knowledge” (p. 1109).

IOM also recognized that AHCs had been slower to adopt LHS. In a 2013 review of the Clinical and Translational Science Awards (CTSA) program, IOM promoted LHS as an important framework for integrating research and practice, which in turn would produce more efficient and effective clinical care (IOM, 2013, p. 36).^11^ IOM encouraged the National Center for Advancing Translational Sciences (NCATS) to prioritize LHS in its management of the CTSA program, for example, by requiring funded institutions to collaborate more actively with practice‐based research networks and to strengthen their community‐engagement efforts (pp. 72‐73).^11^

NCATS responded to the IOM report by incorporating LHS into the next CTSA Funding Announcement (RFA‐TR‐14‐009)^2^ and then added IOM's definition of LHS to the 2015 funding announcement (PAR‐15‐304).^12^ However, these Funding Announcements framed LHS not as a means of promoting organizational learning and improvement of clinical practice, but rather as a concept that supports the scientific goal of increasing the number of patients who are enrolled in clinical trials. For example, the 2014 Funding Announcement included the following language:

With the concept of a learning healthcare system in mind, the applicant should describe system‐wide approaches on how patients receiving care at the hub will be made aware of ongoing research, and invited to partake in research opportunities.

Similar statements were included in the 2015^12^ and 2018^13^ Funding Announcements. Subsequent Funding Announcements made no mention of LHS.

Despite the lack of a clear mandate from NCATS (and NIH more generally), a growing number of AHCs have adopted LHS as a framework. Institutions such as Vanderbilt Medical Center, the University of Alabama at Birmingham and the University of Wisconsin have centers, institutes or departments that focus explicitly on LHS work.^14^, ^15^, ^16^ Atrium Health Wake Forest Baptist (AHWFB) adopted LHS as the theme for its CTSA strategy in its funded 2018 proposal. Moreover, the larger health system (Atrium Health) branded itself as an “Academic Learning Health System” in 2021.^17^ At other institutions, LHS has been adopted more narrowly by individual investigators who use it as a frame for specific learning‐oriented projects or lines of work.^18^, ^19^, ^20^

## 2 MOVING FROM ADOPTION TO IMPLEMENTATION

IOM purposely defined LHS in broad terms so that it could be applied in many different contexts.^5^ This lack of specificity means that any health system that adopts LHS faces the challenge of determining what exactly this will mean in terms of new work, changes in practice, budgeting, staffing, new policies, and changes in culture. This operationalization challenge applies to AHCs just as it does to any other health center.

To guide health system leaders in meeting that challenge, our team conducted a scoping review of scientific publications that described the work that health systems are carrying out, or should be carrying out, under the rubric of LHS.^21^ In that review, we conducted a qualitative analysis of 79 relevant publications, 13 of which focused specifically on LHS in the context of an AHC (eight unique AHCs). We identified 25 “forms of LHS work” that were referenced in at least five publications, which were classified into five “bodies of LHS work”: (a) organizational learning and continuous quality improvement; (b) translating knowledge and evidence into practice; (c) building new knowledge and evidence; (d) analyzing clinical data for learning; and (e) engaging clinicians, patients, and others stakeholders.

AHCs routinely excel at some forms of LHS work identified in the scoping review, especially within the “building new knowledge and evidence” body of work. Indeed, the following two are core to the mission of academic research enterprises:

1. The organization conducts rigorous research that answers questions regarding health and health care that are critical *at a general level*, and
2. Generalizable findings are disseminated to external audiences through publications and scientific presentations.

However, other forms of LHS work are less consistently performed within AHCs, and even may be viewed as misaligned with the mission of an academic research enterprise. Based on our experience at AHWFB and our interactions with other AHCs (especially within the CTSA network), we have found that the following categories of LHS work are particularly challenging to implement within this setting:

1. Conducting studies that focus explicitly on critical patient‐care issues facing the health system itself (as opposed to larger research questions);
2. Conducting studies where clinicians and system leaders are directly involved in specifying research questions, designing studies, and formulating the interventions to be tested;
3. Conducting studies that have short turnaround times in providing answers to health system leaders;
4. Translating study findings into practice changes within the clinical enterprise (as opposed to simply publishing findings in the scientific literature);
5. Engaging patients, families, and other stakeholders in designing and carrying out studies, as well as interpreting and translating the results.

We believe that these five forms of work are under‐emphasized in AHCs because they fall to the periphery of the scientific paradigm, which in turn serves as the frame of reference for conducting research within AHCs.

## 3 CONTRASTING PARADIGMS

In some respects, the LHS paradigm represents a qualitative expansion of the scientific paradigm. For example, the LHS paradigm calls not only for the creation and dissemination of empirical research findings, but also intentional efforts to translate those findings into improved practices. In addition, the LHS paradigm values a broader array of research methodologies, including quality improvement studies and natural experiments which look at changes in outcomes before vs after an intervention is introduced, often without the benefit of a comparison group. Randomized designs are sometimes used in LHS research, but these are generally conducted as pragmatic clinical trials, which means that the interventions being evaluated are carried out under natural conditions within the health system, rather than through tightly‐controlled protocols with careful attention to fidelity.^22^, ^23^, ^24^, ^25^ Because they use random assignment and systematic measurement methods, pragmatic clinical trials produce valid, reliable findings which are publishable within high‐impact scientific journals. At the same time, it is important to point out that the vast majority of biomedical science is conducted in more controlled settings.

In addition to expanding the portfolio of acceptable research methods, the LHS paradigm brings a different orientation in developing the research question. With traditional biomedical science, the research question originates with the principal investigator, typically building directly and incrementally on a long line of research on a defined topic. In addition, scientists emphasize the creation and dissemination of knowledge, often leaving it to others to translate findings into clinical practice. Their perspective is typically at a field level, rather than the clinical enterprise at their own institution. This contrasts with the LHS paradigm, where the research process begins with the identification of a practical issue or problem within clinical care or operations. Leaders within the institution are typically involved in determining which research questions will be answered. In addition, interventions that are studied under the LHS paradigm often emerge from clinical practice and quality improvement initiatives.

Another key distinction between the LHS paradigm and the scientific paradigm involves the timetable for producing results. The LHS paradigm emphasizes timely investigation of clinically relevant questions with adoption of results into the institution's own operations and clinical care. Institutional leaders’ desire for quick answers is incompatible with the lengthy process of securing extramural funding (ie, preparing a proposal, submitting for peer review, waiting for the funding decision, and in most cases, resubmitting based on comments from reviewers). The desire for a quick win also often requires a compromise on study rigor, for example, the use of historical controls or the absence of randomization. Even with internal funding, traditional researchers may be discouraged from leading LHS studies if they perceive it will be difficult to publish their findings.

## 4 SUPPORT FOR LHS RESEARCH WITHIN AHCs

This contrast in paradigms has implications for whether the LHS construct will be embraced (or even accepted) within AHCs, as well as whether researchers will be available to conduct the practical research that the LHS paradigm calls for.

The culture of most AHCs places a high value on scientific achievements, as measured by peer‐reviewed publications, editorial positions, extramural grants (especially from NIH), selection for study sections, and invited seminars. These expectations are explicitly incorporated into the criteria for promotion and tenure. Academic researchers pursue their own scientific agendas and respond to the priorities of the agencies that fund science. They are not waiting for assignments or direction from health system leaders. In addition, they are focused on building their scientific reputation and meeting the expectations for promotion and tenure; “service”‐oriented research that does not result in peer‐reviewed publications is often viewed as a distraction from their career goals. The typical AHC has hundreds or even thousands of PhD‐trained researchers with skills directly relevant to LHS research, but this does not mean they are available to conduct LHS research. This is not to suggest that a researcher cannot simultaneously pursue both LHS and generalizable research, but rather this is the exception rather than the norm within academic medicine.

Another key challenge in implementing the LHS paradigm is the chasm between the research enterprise and the clinical enterprise. Researchers often sit, both functionally and physically, in different parts of the organization than do clinicians. As a result, researchers are often disconnected from their clinical colleagues and not attuned to the issues that most directly affect the health of the system's own patients. This disconnection also limits the innovation and translation that can occur across research and clinical spaces.^10^

## 5 REMEDIES

In order to truly operate as an LHS, an AHC needs to integrate the LHS paradigm into its scientific enterprise. This begins with clear indications from health system leadership that LHS research is valued.^21^, ^26^ One strategy is to encourage and incentivize department‐appointed research faculty to devote a portion of their effort to LHS‐oriented studies that overlap with their own interests. This strategy is typically interpreted as requiring the health system (and more specifically the clinical enterprise) to invest its own funds into research directly aligned with clinical and/or operational priorities. While LHS research typically requires at least some internal investments, national funding agencies such as PCORI and NIH are increasingly making at least some funding available through programs such as PCORI Health System Implementation Initiative, NIH Collaboratory, and NIA Impact Collaboratory.^27^, ^28^, ^29^ These federal programs are valuable not only for their funding, but because they help legitimize LHS as credible science that supports an investigator's scientific reputation, promotion and tenure.

An alternative (or complementary) strategy is to establish an entity dedicated to LHS research which operates in parallel to scientifically oriented departments and centers. Researchers based in this entity embed themselves in the relevant clinical or administrative units to design and conduct a study that balances scientific rigor, timeliness and budget constraints in order to produce reliable and actionable findings. Vanderbilt University Medical Center's LHS Platform, housed within its Institute for Clinical and Translational Research, was established with this purpose in 2016 and has developed a specialty in pragmatic clinical trials.^14^ This strategy replicates the embedded research models that were pioneered by Kaiser Permanente,^30^ Group Health (now Kaiser Permanente Washington),^6^ and Atrium Health.^31^

These two approaches offer a path forward for implementing the LHS paradigm within AHCs, but not without challenges. With the first strategy of integrating LHS research directly into the research enterprise, a key task is to identify and incentivize faculty who are interested in conducting studies that address the practical issues facing the health system. Researchers with this phenotype need to be linked to projects that intrigue them and their effort needs to be covered with institutional funding so that they are not penalized for taking time away from extramurally funded research. This funding needs to be provided over the lifespan of the project, beginning with the initial stage of building relationships with collaborators.

Under either approach, there is also the question of whether researchers who conduct LHS research will fit and thrive within the context of academic research. Because LHS studies balance rigor with practicality and timeliness, they tend to produce fewer publications, especially in high‐impact journals, which can have negative consequences when it comes to promotion and tenure. One remedy is for LHS researchers to rely more heavily on pragmatic clinical trials (with random assignment). This methodology generates study findings that contribute to science, while also being locally relevant.

It is also important for the promotion process to recognize that the LHS paradigm can produce more relevant science and greater impact. These benefits stem in large part from the LHS paradigm's emphasis on researchers collaborating with practicing clinicians to address real‐world issues. These partnerships allow researchers to develop more informed and relevant research questions. In addition, their experimental interventions and study protocols can be improved by incorporating lessons from CQI initiatives. And partnering with clinicians lays the groundwork for study findings to be translated into practice.

## 6 CONCLUSION

The LHS concept promoted by NAM represents a bold innovation that combines organizational learning, strategic analysis of patient data, stakeholder engagement and the systematic translation of research into practice—all in service of improving the quality of health care delivered across the organization. This innovation has been diffused and widely adopted by healthcare organizations over the past 15 years, but AHCs have been slower on the uptake.

The growing number of AHCs that are adopting the LHS paradigm and identifying themselves as “learning health systems” suggests there can be alignment between LHS and academic medicine. However, this requires careful attention to the “enabling conditions” that support the adoption of the LHS paradigm, including a workforce skilled in LHS competencies, data systems and informatics infrastructure, dedicated resources invested in LHS work, and a supportive organizational culture.^21^ AHCs generally are well endowed in the first two of these conditions, but are more variable when it comes to investing funds in LHS research and expressing a clear institutional commitment to the LHS paradigm.

Committing to the LHS paradigm is inherently easier for an AHC's clinical enterprise than it is for the research enterprise. Clinicians and clinical leaders gain direct benefits from LHS research (eg, learning whether an innovation in care delivery is effective, gaining deeper knowledge about problems such as hospital‐acquired infections). Leaders of the research enterprise tend to be more resistant to the LHS paradigm because of its emphasis on applied research; generalizable knowledge is a collateral benefit, rather than the defining purpose.

The defining challenge in extending the LHS paradigm to AHCs is that LHS appears to be misaligned with one of their three missions—that of the research enterprise. Nine years ago, Grumbach et al. proposed a remedy that involves AHCs integrating their separate research, clinical and educational missions into a single LHS‐oriented mission emphasizing the “synergistic cycle of discovery, education and care delivery.” Limited progress has been made on this bold proposal. To make a meaningful change, AHCs need to demonstrate a tangible commitment to the LHS, including dedicated funding for LHS work and promotion criteria that reward LHS research.

## CONFLICT OF INTEREST STATEMENT

The authors have no conflicts of interest to report.
